# Prevalence of Pre-Eruptive Intracoronal Resorption (PEIR) and Proposal of a Novel Classification: Retrospective Study with the Aid of Cone Beam Computed Tomography (CBCT)

**DOI:** 10.3390/dj14020118

**Published:** 2026-02-17

**Authors:** Emmanuel Mazinis, Konstantinos Ioannidis, Shanon Patel, Vassilis Karagiannis, Christos Gogos

**Affiliations:** 1 Department of Endodontology, Dental School, Aristotle University of Thessaloniki, 541 24 Thessaloniki, Greece; konioannidis@dent.auth.gr (K.I.); gogos@dent.auth.gr (C.G.); 2 King’s College, Guy’s Campus, Great Maze Pond, London SE1 1UL, UK; shanon.patel@kcl.ac.uk; 3 School of Mathematics, Aristotle University of Thessaloniki, 541 24 Thessaloniki, Greece; vkdstat@math.auth.gr

**Keywords:** cone beam computed tomography, impacted teeth, pre-eruptive intracoronal resorption, resorption, unerupted teeth

## Abstract

**Background/Objectives**: Pre-eruptive intracoronal resorption (PEIR) in impacted or unerupted teeth often remains undiagnosed. The aim of this study was to investigate the prevalence of PEIR with the aid of cone beam computed tomography (CBCT) and propose a new three-dimensional (3D) classification for the analysis of the lesions. **Methods**: A total of 164 unerupted teeth diagnosed in CBCT scans, derived from an equivalent number of patients, were examined for the presence of PEIR, tooth type, angulation and position. A novel 3D classification system was proposed and all PEIR lesions were further classified. The classification system was used to stage PEIR lesions according to their extend from the enamel level apically, the circumferential spread and their proximity to the pulp chamber. Descriptive statistics were used to assess the prevalence and type of resorption. The association between PEIR, demographics, tooth type, position and angulation were studied. The estimation of the multivariate relationship between PEIR, patient’s demographics and tooth characteristics was conducted with the multiple binary logistic regression model. **Results**: The prevalence of PEIR was 33.5%, affecting mostly maxillary canines, and maxillary and mandibular molars. The prevalence of PEIR in ages over 45 years was significantly higher (*p* < 0.001). The presence of PEIR was significantly associated with buccal position (*p* = 0.002) and buccal angulation (*p* = 0.016) of the tooth. **Conclusions**: Due to the high prevalence of PEIR, CBCT may improve detection and 3D characterization when imaging is already clinically indicated, and influence treatment planning in selected cases.

## 1. Introduction

Root resorption develops as a result of physiologic or pathologic loss of hard dental tissues, including dentin and cementum [1]. Broadly, there are two categories of resorption: external and internal root resorption. External root resorption manifests in various forms depending on histological and radiographic criteria, including surface, inflammatory, replacement, cervical and transient apical root resorption [1,2,3,4]. External cervical resorption (ECR) is a destructive process, which usually starts in the cervical region of the tooth, resulting in progressive loss of cementum and dentin by odontoclasts that resorb the dental hard tissue [3,4].

Cone beam computed tomography (CBCT) improves the detection of ECR compared to conventional radiographs [5,6,7]. CBCT has been advocated by both the AAE and the ESE for the management of ECR [8,9]. Patel et al. proposed a three-dimensional (3D) classification for ECR, based on CBCT findings, assessing lesions by height, circumferential spread, and proximity to the pulp [10]. This classification is effective for accurate diagnosis, and description of the extent and severity of the lesion.

ECR in impacted or unerupted teeth is also referred to as pre-eruptive intracoronal (PEIR) or intramural resorption [11,12,13,14]. Pre-eruptive intracoronal resorption (PEIR) is a rare condition characterized by a well-defined radiolucent lesion within the pulpal or root dentine of impacted or unerupted teeth as a result of dental hard tissue breakdown [15]. It is typically detected incidentally in panoramic (OPG) or bitewing (BW) radiographs with a tooth prevalence of 0.2–3.5% in permanent mandibular and maxillary molars respectively [16]. The histological findings of PEIR correspond to those of ECR, as in most case studies, the lesions consist of similar features, including inflammatory resorptive cells without evidence of microbial invasion, caries or pulp degeneration [17].

The two-dimensional observation of teeth and potentially existing lesions, as well as the inherent geometric distortion of such images, prompts its limited capacity to detect PEIR in impacted or unerupted maxillary teeth compared to mandibular ones [13]. The diagnostic limitations of detecting PEIR are further subjected to the orientation and topography of the lesion, particularly if they do not manifest in the mesio-distal axis or if they are not located in the labial aspect.

Despite the aforementioned limitations of two-dimensional OPG and BW, the proposed classification by Seow et al., comprising severity scores 1–3 [Score 1: within one third dentin thickness; Score 2: two-thirds dentin thickness; and Score 3: extended through the full dentin thickness of the crown], has been widely adopted [11,16,18]. Yuksel et al. proposed an advanced fifteen-grade scoring system for the detection of PEIR, but it was still based on OPG [19]. Recent studies have highlighted that the use of CBCT improves accuracy in the detection of PEIR and provides a more detailed three-dimensional description of the resorptive lesions [12,14,20].

The prevalence of ECR has been reported to be 1 to 2.3%; however, this is most probably an under-estimation as these studies are based on small field of view CBCT scans only [21,22]. The application of CBCT for the detection and prevalence calculation of PEIR was implemented in the retrospective studies of Demirtas et al. and Ngamsom et al. to overcome the limitations of two-dimensional imaging [12,14]. Despite the acknowledgement of the superior potential from the use of CBCT, they continued implementing the 1–3 scoring system and minor modifications, including a fourth and fifth grade of severity. However, no attempt was made to adjust their observations into a 3D classification system, with a view to adequately assess the 3D nature of PEIR. Therefore, it is well documented that PEIR in unerupted or impacted teeth is not included yet in the classification systems [23]. A new classification system is required to tighten the gap in international guidelines to encompass conditions involving PEIR similar to external cervical root resorption (ECR), as both share common histological and radiographic characteristics.

The aim of the study was to investigate the prevalence of PEIR on impacted and unerupted teeth with the aid of CBCT, utilize data from the 3D evaluation and propose an international classification to describe the presence of external invasive resorptive lesions in impacted or unerupted teeth.

## 2. Materials and Methods

The retrospective study involved the evaluation of CBCT data obtained from dental patients referred to a private dental radiology center, in the province of Central Macedonia, Veroia, Greece, between September 2018 and February 2024, for the diagnostic justification of dento-alveolar and/or orofacial disease. All patients provided written informed consent for the CBCT examination at the time of imaging. Ethical approval for the study was obtained from the Bio-Ethics Committee of the School of Dentistry, Aristotle University of Thessaloniki, Greece (Date 13 January 2025 meeting with protocol number 26/29-01-2025). The retrospective research analysis used anonymized data; the ethics committee approved the study and waived the requirement for additional consent for retrospective use.

Scans were acquired using a NewTom CBCT (Cefla Group, Imola, Italy) unit under high-resolution settings (90 kVp, 4 mA, 0.1 mm voxel size, and 360° rotation), in accordance with the ALARA (As Low As Reasonably Achievable) principles.

Eligible individuals from the study population were aged between 22 and 83 years. Patients younger than 22 were excluded to ensure that the examined teeth had completed their pre-eruptive development. A tooth was defined as impacted when it was covered by bone and/or mucosa and remained below the occlusal plane. Patient demographic data, including age and sex, along with the characteristics of the impacted teeth (tooth type, position, and angulation), were recorded.

All patients had non-contributory medical histories, with no systemic conditions (e.g., hyperparathyroidism or Paget’s disease, developmental dental anomalies, unerupted teeth associated with cysts, and benign or malignant tumors) known to affect dental hard tissue resorption. Furthermore, images characterized by inadequate quality, blurring beam-hardening artifacts or other severe artifacts were excluded from the analysis. Τhe minimum lesion size was set at >1 mm, requiring visibility in ≥2 planes.

A total of 1237 CBCT scans were examined for the presence of at least one impacted tooth. Only scans with exactly one impacted/unerupted tooth were eligible; scans with multiple impacted/unerupted teeth were excluded. From these, *n* = 164 scans fulfilled the predefined criteria and were included in the study. All scans were obtained from distinct patients. The type, angulation, and position of each impacted tooth were evaluated. Tooth angulation and position were categorized as mesio-angular, disto-angular, vertical, horizontal, bucco-version, linguo-version, or inverted.

The CBCT scans were assessed by three independent observers [EM, SP, KI], each with over 15 years of experience using NNT Viewer software (Cefla Group, Imola, Italy), in cross-sectional and multiplanar views (slice thickness: 0.125 mm), under standardized office and light conditions, on the same monitor (1350:1 contrast ratio) (EIZO 24″ MX243W RadiForce, (EIZO Corporation, Hakusan, Japan). The observers were able to enhance the images freely by adjusting the radiographic contrast, brightness and magnification.

The presence of PEIR lesions was documented and compared among all three observers to ascertain the prevalence of lesions. Intra-examiner reliability was assessed over a period of 4 weeks, and any discrepancies were resolved through a consensus meeting among all observers involved in this study.

**Figure 1:**
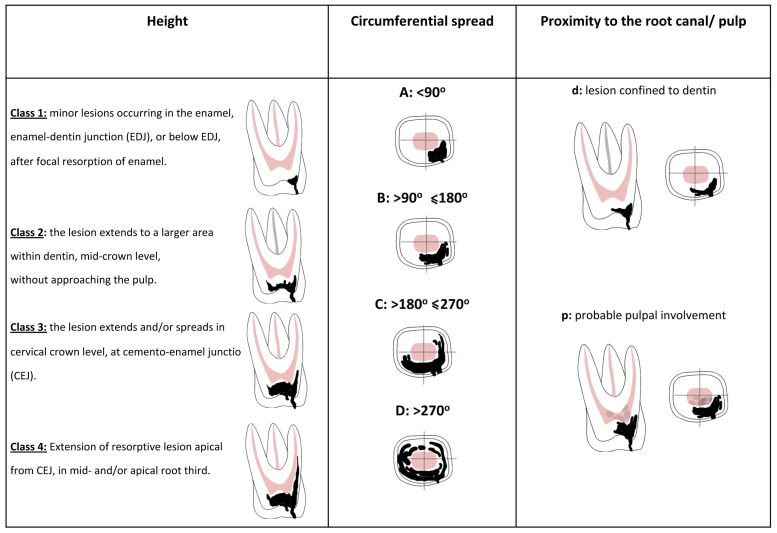
The novel classification system of PEIR based on 3D assessment of lesion size and probable pulpal involvement.

**Figure 2:**
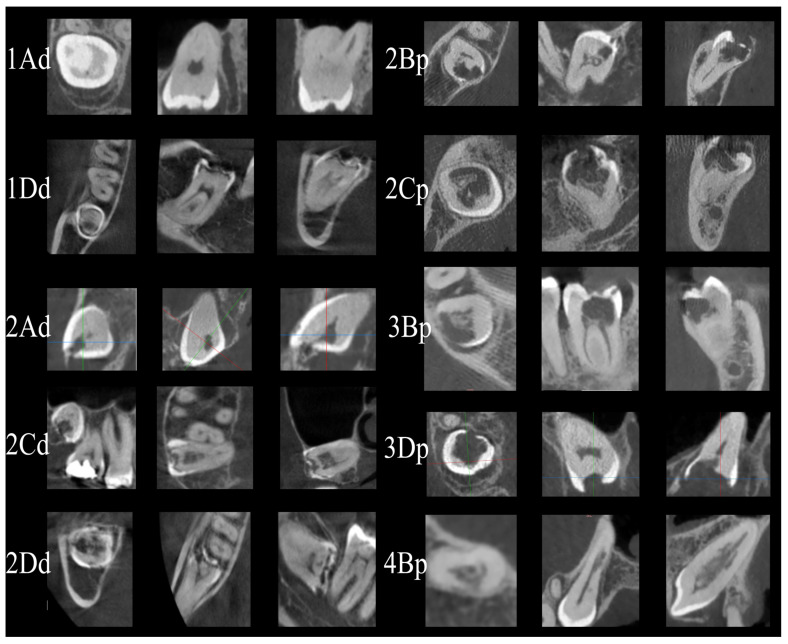
CBCT images of various types of PEIR according to novel classification.

In cases where a resorptive lesion was present, a modified classification of PEIR lesions was employed. The description and assignment of the degree of resorption was based on a modified version of the three-dimensional scoring system originally proposed by Patel et al. (2018), which was originally used to assess the staging of cervical root resorption in erupted teeth [10] [Figure 1]. This CBCT-based classification system is appropriate for the detailed assessment of lesion size, extent, and location in three dimensions in unerupted teeth, providing a more accurate description of the lesion size compared to two-dimensional categorizations. The modified classification was used to stage lesions extending from the enamel to an apical extend (height), according to the circumferential spread (0–360°) and proximity to the pulp chamber [Figure 2].

The height (coronal–apical extent) of the lesion is graded according to its maximum vertical extension from the crown level to the cemento-enamel junction (CEJ) and apically within the root surface [coronal, middle or apical root third]. The height of the lesion can be best assessed by using the coronal and sagittal CBCT views.

Class 1: Minor lesions occurring in the enamel, enamel–dentin junction [EDJ], or below the EDJ, after focal resorption of enamel.
Class 2: The lesion extends to a larger area within the dentin, mid-crown level, without approaching the pulp.
Class 3: The lesion extends and/or spreads within the cervical crown level, at the cemento-enamel junction (CEJ).
Class 4: Extension of the resorptive lesion apical from the CEJ, in the mid- and/or apical root third.

The circumference of the lesion is graded according to its maximum spread within the crown or root surface and it is best assessed using axial CBCT views.

A: <90°
B: >90° ≤ 180°
C: >180° ≤ 270°
D: >270°

The proximity of the lesion to the pulp chamber or the root canal can be best assessed using axial CBCT views.

d: Lesion confined to dentine.
*p*: (Probable) pulpal involvement.

## 3. Statistical Analysis

Regarding the presence of PEIR, intra-rater reliability for each of the three observers across all included CBCT scans (*n* = 164) was assessed using Cohen’s kappa and demonstrated perfect agreement (k = 1.00 for all observers). Inter-rater reliability across all scans was evaluated using Fleiss’ kappa for multiple observers, yielding a value of k = 0.991 (95% CI: 0.902–1.000), before any consensus discussion. Descriptive analyses were performed to assess the prevalence of resorption both in the patients’ study population and the population of teeth, as well as the distribution of classification levels within each tooth type. Associations between resorption and demographic variables, including age (categorized into five groups) and gender, as well as associations between resorption and tooth-related characteristics (tooth type, position, and angulation), were examined using Fisher’s exact test for variables with two categories or the chi-square test for variables with more than two categories. For chi-square analyses, results were considered reliable when the expected frequency in each cell exceeded one; otherwise, *p*-values and their corresponding 99% confidence intervals were estimated using Monte Carlo resampling (20,000 iterations). The multivariable association between resorption and patient demographics and tooth-related characteristics was evaluated using multiple binary logistic regression. Odds ratios were reported with 95% confidence intervals. All statistical analyses were performed using the R statistical software (R Version 4.5.2, RStudio) integrated development environment). The stats package was used for binary logistic regression, coin and vcd for Fisher’s exact and chi-square tests with or without Monte Carlo resampling, and irr for reliability and agreement analyses. Statistical significance was set at *p* < 0.05 for all tests.

## 4. Results

**Figure 3:**
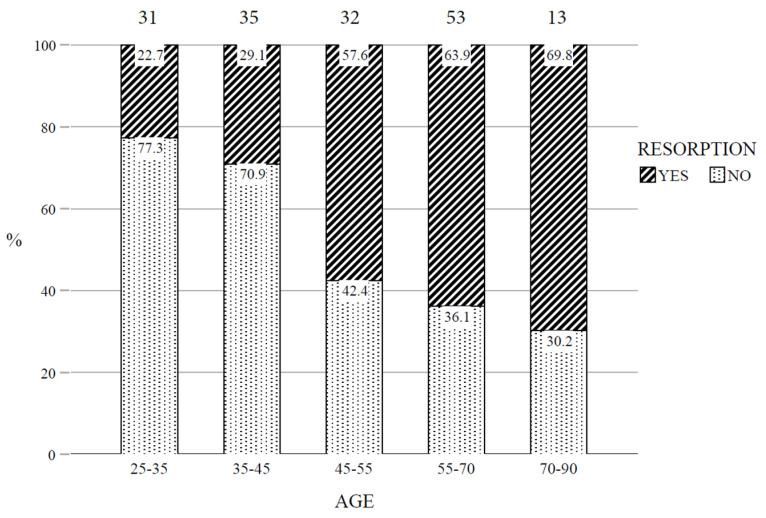
Prevalence of RESORPTION within AGE categories, *n* = 164 cases (the total of each category is included over the corresponding bar).

The 164 studied scans (one scan per patient) originated from 91 males, aged from 25 to 82, with an average age of 47.8 years and 73 females, aged from 25 to 83, with an average age of 52 years. The prevalence of PEIR, between 25 and 45 years, was low (12.9% and 17.1% respectively), while the prevalence was significantly higher in patients who were 45+ years (*p* < 0.001) [Figure 3]. The prevalence of PEIR was found to be 40.6% between 45 and 55 years, 47.2% between 55 and 70 and 53.8% between 70 and 90 years. Accordingly, in the multiple logistic regression model, age was classified into two categories, less than 45 and at least 45.

With respect to gender, although a higher prevalence of resorption was observed in females than in males (41.1% − 27.5% = 13.6%), the difference did not reach statistical significance (*p* = 0.066) [Table 1].

**Figure 4:**
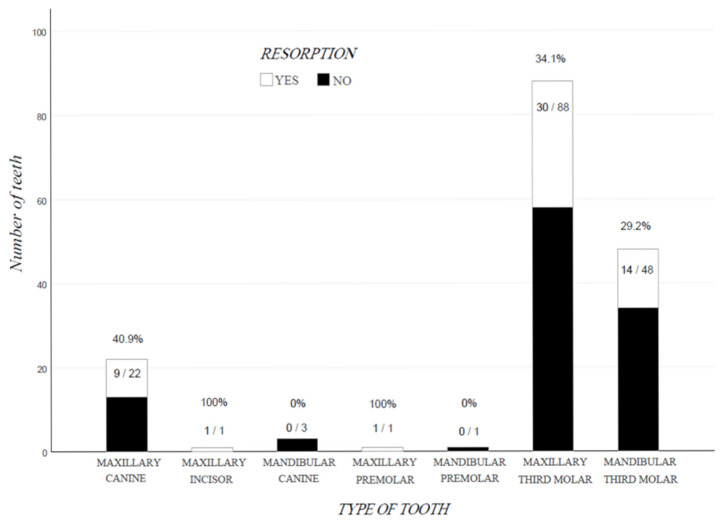
Prevalence of RESORPTION for each TYPE OF TOOTH, *n* = 164 cases. Over or inside each bar, x/y means x cases with RESORPTION, y cases without RESORPTION, while the given percentage is calculated by the form 100x/(x + y).

No statistically significant association was observed between resorption and type of tooth (*p* = 0.326), however PEIR most commonly affected canines (40.9%), maxillary third molars (34.1%) and mandibular third molars (29.2%) [Figure 4].

Statistically significant results were found for tooth position, where the prevalence of the resorption in the buccal position (69.6%) was found to be more than two times greater than the corresponding prevalence values in central (25.9%) and lingual/palatal (30.4%) [Table 1] positions. Moreover, the association between resorption and tooth angulation was also significant, with buccal orientated teeth showing the greater percentage of resorption cases (68.4%; almost two times greater than the other categories except for the category linguo-version for which the prevalence was equal to 50%) (*p* = 0.016) [Table 1].

The prevalence of the proposed 11 classification levels of resorption within each one tooth type is presented in Table 2. Taking into account the small sample size compared to the number of classification levels, it is worth noting the high prevalence value of the IAd level (21.6% or 19/88 teeth) in the maxillary third molars, whereas in almost all the other tooth types most of the classification levels were observed with small, nearly homogeneous values (from 1.1% to 9.1%). Furthermore, no resorption was observed in mandibular canine and mandibular premolar tooth categories.

In Table 3, the results of the multiple binary logistic regression model are presented. When all other variables are kept constant, gender is not significant at the 5% level but is significant at the 10% level of significance (*p* = 0.065). Keeping all other variables constant, the prevalence of resorption in ages 45 and older is four times higher in individuals less than 45 years (OR = 4.036, 95% CI: 1.787–9.115, *p* < 0.001), while the prevalence of resorption in types bucco-linguoversion are expected to be three times higher than in types mesio-disto angular (OR = 3.194, 95% CI: 1.149–8.877, *p* = 0.026).

## 5. Discussion

ECR in erupted teeth as well as PEIR in unerupted teeth are usually asymptomatic and diagnosed as incidental findings [2,23]. Histological differences in the enamel structure between erupted and unerupted teeth are well documented [24]. Crown impaction may predispose progressive enamel resorption [25]. Genetic, developmental or pathological conditions can further result in hypomineralization and enamel hypoplasia, which may contribute to or be associated with the resorptive process [26]. A correlation has been found between patient age and dental follicle inflammation as the longer the follicular tissue remains embedded in the bone, the higher the probability of an inflammatory reaction developing in the connective tissue [27].

In unerupted teeth, some potential predisposing factors associated with ECR such as trauma, history of orthodontic treatment, occlusal disorders or periodontal disease are absent. However, age-related changes in the dental follicle may further combine with close proximity to surrounding inflamed areas [e.g., deep periodontal pockets, retained deciduous teeth, endodontic lesions or sinus infections]. Additionally, increased pressure on unerupted teeth may cause the reduced enamel epithelium to differentiate, potentially activating resorptive cells that initiate dentin resorption [15]. Hypoxia may pose a triggering effect for the initiation of root resorption [28]. It remains unclear whether unerupted teeth are affected through similar mechanisms to those observed under hypoxic conditions induced by orthodontic forces.

In our study, a total of 164 impacted and unerupted teeth, from 164 patients, were evaluated. Among these, 55 teeth exhibited PEIR, which was a prevalence of 33.5% (55/164). The likelihood of resorption increased with age, indicating the progressive nature of the phenomenon, with a significantly higher prevalence observed in the age group of 45–55 years and the focal entry of resorption was detected on all surfaces of impacted or unerupted teeth. Although no statistically significant association was found between resorption and the type of tooth, relatively high prevalence values were observed for maxillary canines, and mandibular and maxillary third molars. Ngamsom et al. detected a higher resorption rate (83%) and the angulation was not found to be statistically related to increased or decreased prevalence of PEIR [14]. In this study, specific characteristics of unerupted teeth, including their angulation and spatial orientation, showed a clear correlation with the prevalence of PEIR, as bucco-linguoversion was three times more frequently associated with resorption than mesio-distal angulation.

An issue concerning the methodology of the study, as well as the definition of an unerupted tooth, relates to the age groups included. The use of the term PEIR in younger age groups clearly refers to lesions in teeth that have not yet erupted, but their eruption may still occur before adulthood. The inclusion of mixed age groups may lead to confusion regarding the definition of PEIR and, consequently, affect statistical analysis.

Earlier studies using panoramic (OPG) and bitewing (BW) radiographs in pediatric populations showed a limited diagnostic value for detecting PEIR due to geometric distortion and anatomical noise [16,20,29]. More recent investigations have used CBCT in both adult and non-adult populations [12,14,30], but none applied a 3D classification system to characterize resorptive defects. The existing ranking systems rely solely on the appearance of a lesion in two dimensions, without further three-dimensional assessment [14,16,17,20,31].

Therefore, one of the main objectives of this study was to bridge the existing gap of the 3D observation and the descriptive analysis of the lesion in three topographic co-ordinates, including height, circumferential spread and proximity to the pulp. Hence, a novel modification of the existing ECR classification index was proposed.

Several studies attempted to calculate the overall prevalence of resorption in population samples [11,16,29,32]. The prevalence of PEIR reported by CBCT studies [12,14] was lower compared to the present study. Demirtas et al. reported a prevalence of PEIR in 15.1% of teeth, with a higher frequency in mandibular and maxillary molars [12]. Ngamsom et al. assessed 380 patients with 590 unerupted teeth in patients aged 7–69 years, and reported resorption in 76 patients (32 males and 44 females), with a subject prevalence of 20% [14]. In total, 80 teeth exhibited resorption, resulting in a tooth prevalence of 13.6%. It is important to note that the sample population in both studies included both adult and non-adult populations [12,14]. Although a study based on OPG analysis reported no significant statistical differences in the occurrence of resorption between adult and non-adult populations, our findings indicate that age influences both the onset and the progression of resorption 32. Hence, the exclusion of non-adult patients may improve the consistency and reliability of the analysis, as resorption may have not yet developed or progressed to detectable levels. Therefore, selecting adult patients ensures that all teeth, including third molars, can reasonably be considered unerupted, thereby avoiding misclassification.

The occurrence of external invasive resorption in unerupted teeth [PEIR] has not yet been categorized as a distinct clinical entity. The following criteria may be considered in the diagnosis of PEIR, including: [a] the use of CBCT for the detection and classification of the lesion; [b] the presence of a focal surface defect; [c] the presence of an intact predentin layer of the pulp; and [d] the histopathological findings [23].

In clinical practice, PEIR should be easily differentially diagnosed from caries, due to the location of unerupted teeth and the lack of crown exposure in the oral cavity. In addition, early diagnosis of PEIR is contributory to treatment planning and risk factor management, especially in orthodontics when impacted canines require intervention, surgical exposure and orthodontic alignment. Particularly, considering the high prevalence of PEIR in impacted maxillary canines, the use of CBCT enables the clinicians to inspect tooth structural integrity and suitability for further treatment.

Surgical extraction of such teeth, when indicated, is more complex, due to the risk of crown fracture and the potential ankylosis that may co-exist in such cases. Finally, the early detection and diagnosis of PEIR is essential as far as tooth re-implantation or transplation procedures are concerned. Following tooth loss after trauma and especially in young ages, apart from the close inspection of external root and crown anatomy of candidate teeth for extraction and re-implantation, it is critical to inspect impacted teeth for the presence of PEIR. It remains unknown whether a transitional biological process from PEIR to ECR may exist, soon after a tooth is transplanted in a functional position, intra-orally. Hence, the existence of undetected PEIR may compromise treatment outcomes, which in principle are of a complex nature.

Referral patterns for CBCT serve as primary determinants of a study’s external validity, specifically its population validity, and if they are concentrated in specialized settings—such as university-based hospitals or oral surgery centers—the resulting research samples may not be representative of the broader patient population seen in general private practice. In this study, the selection of the only available private radiology referral center was appropriate to serve as the study population.

A limitation of the present study is the sample size, acquired from a specific geographic province. Hence, it cannot account for the potential trends in the general population. Further studies on a wider geographic scale, or in populations of different ethnicities may allow for more conclusive outcomes regarding the resorption classification and reporting scores. Another limitation is the limited capacity to obtain histological data and study or further confirm the nature of the resorption. Although studies in which the impacted tooth was extracted support its resorptive nature, this cannot be documented within a systematic review.

## 6. Conclusions

The occurrence of PEIR in impacted and unerupted teeth is higher than is commonly perceived. CBCT may improve detection and 3D characterization when imaging is already clinically indicated, and may influence treatment planning in selected cases (e.g., impacted canine management). The proposed 3D classification enables the early detection of PEIR in impacted and unerupted teeth and may critically affect decision-making in restorative, orthodontic and surgical treatment planning.

## Figures and Tables

**Table 1:** The frequency and distribution of resorption within patients’ demographics as well as within the tooth characteristics tooth type, position and angulation and the corresponding *p*-values (exact, asymptotic or with Monte Carlo resampling) from the association tests between resorption and each one of the mentioned variables.

		Resorption				
		YES		NO		
		*n*	%	*n*	%	*p*-Value
Age	25–35	4	12.9	27	87.1	
	35–45	6	17.1	29	82.9	
	45–55	13	40.6	19	59.4	
	55–70	25	47.2	28	52.8	
	70–90	7	53.8	6	46.2	0.001 **^a^**
Gender	Male	25	27.5	66	72.5	
	Female	30	41.1	43	58.9	0.066 **^b^** (*NS*)
Type of tooth	Maxillary Canine	9	40.9	13	59.1	
	Maxillary Incisor	1	100.0	0	0	
	Mandibular Canine	0	0	3	100.0	
	Maxillary Premolar	1	100.0	0	0	
	Mandibular Premolar	0	0	1	100.0	
	Maxillary Third Molar	30	34.1	58	65.9	
	Mandibular Third Molar	14	29.2	34	70.8	0.292 (0.284–0.301) **^a,c^** (*NS*)
Position	Central	29	25.9	83	74.1	
	Buccal	16	69.6	7	30.4	
	Lingual/Palatal	7	30.4	16	69.6	
	Mesial	1	50.0	1	50.0	
	Distal	2	50.0	2	50.0	0.001 (0.0005–0.0017) **^a,c^**
Angulation	Mesioangular	14	25.9	40	74.1	
	Distoangular	6	31.6	13	68.4	
	Horizontal	5	38.5	8	61.5	
	Vertical	10	23.3	33	76.7	
	Buccoversion	13	68.4	6	31.6	
	Linguoversion	6	50.0	6	50.0	
	Inversion	1	25.0	3	75.0	0.016 **^a^**

^a^: Chi-square test, ^b^: Fisher’s exact test, ^c^: *p*-value (99% CI) from Monte Carlo resampling. *NS*: Not significant.

**Table 2:** Prevalence of CLASSIFICATION levels within each type of tooth (cases which were classified ‘NO RESORPTION’ are not displayed).

TYPE OF TOOTH	CLASSIFICATION												
		IAd	IDd	IIAd	IIBd	IIBp	IIDp	IIIBp	IIICp	IIIDp	IIIDd	IVBp	Total
Maxillary Canine	N	1	0	2	1	0	0	0	1	1	1	2	22
	%	4.5%	0.0%	9.1%	4.5%	0.0%	0.0%	0.0%	4.5%	4.5%	4.5%	9.1%	100%
Maxillary Incisor	N	0	0	0	1	0	0	0	0	0	0	0	1
	%	0%	0%	0%	100%	0%	0%	0%	0%	0%	0%	0%	100%
Mandibular Canine	N	0	0	0	0	0	0	0	0	0	0	0	3
	%	0%	0%	0%	0%	0%	0%	0%	0%	0%	0%	0%	100%
Maxillary Premolar	N	1	0	0	0	0	0	0	0	0	0	0	1
	%	100%	0%	0%	0%	0%	0%	0%	0%	0%	0%	0%	100%
Mandibular Premolar	N	0	0	0	0	0	0	0	0	0	0	0	1
	%	0%	0%	0%	0%	0%	0%	0%	0%	0%	0%	0%	100%
Maxillary Third Molar	N	19	0	1	2	1	0	1	2	4	0	0	88
	%	21.6%	0.0%	1.1%	2.3%	1.1%	0%	1.1%	2.3%	4.5%	0%	0%	100%
Mandibular Third Molar	N	4	1	1	0	1	2	0	1	4	0	0	48
	%	8.3%	2.1%	2.1%	0%	2.1%	4.2%	0%	2.1%	8.3%	0%	0%	100%
**Total**	N	25	1	4	4	2	2	1	4	9	1	2	164
	%	15.2%	0.6%	2.4%	2.4%	1.2%	1.2%	0.6%	2.4%	5.5%	0.6%	1.2%	100%

**Table 3:** Odds ratios, with 95% CI, that resulted from the multiple binary logistic regression model with RESORPTION as the response and patients’ demographics and tooth characteristics as the predictors.

	B	S.E.	Wald	df	Odds Ratio (OR)	95% C.I. for OR		
						Lower	Upper	*p*-Value
**GENDER**								
Female					1	Reference		
Male	−0.699	0.379	3.410	1	0.497	0.237	1.044	0.065
**AGE**								
Less than 45					1	Reference		
45 and older	1.395	0.416	11.264	1	**4.036**	**1.787**	**9.115**	**<0.001**
**TOOTH TYPE**								
Posterior					1	Reference		
Anterior	0.452	0.558	0.657	1	1.571	0.527	4.688	0.418
**ANGULATION**								
Mesio-disto angular					1	Reference		
Horizontal-vertical	0.099	0.429	0.053	1	1.104	0.476	2.562	0.817
Bucco-linguoversion	1.161	0.522	4.956	1	**3.194**	**1.149**	**8.877**	**0.026**
Inversion	−0.890	1.604	0.308	1	0.411	0.018	9.519	0.579
**POSITION**								
Buccal-lingual					1	Reference		
Central	−0.734	0.495	2.197	1	0.480	0.182	1.267	0.138
Mesial-distal	0.568	1.289	0.194	1	1.765	0.141	22.069	0.659

## Data Availability

The original contributions presented in this study are included in the article. Further inquiries can be directed to the corresponding author.
